# Skeletal myotube-derived extracellular vesicles enhance itaconate production and attenuate inflammatory responses of macrophages

**DOI:** 10.3389/fimmu.2023.1099799

**Published:** 2023-03-02

**Authors:** Atomu Yamaguchi, Noriaki Maeshige, Jiawei Yan, Xiaoqi Ma, Mikiko Uemura, Mami Matsuda, Yuya Nishimura, Tomohisa Hasunuma, Hiroyo Kondo, Hidemi Fujino, Zhi-Min Yuan

**Affiliations:** ^1^ Department of Rehabilitation Science, Kobe University Graduate School of Health Sciences, Kobe, Japan; ^2^ School of Life Sciences and Technology, ShanghaiTech University, Shanghai, China; ^3^ Graduate School of Science, Technology and Innovation, Kobe University, Kobe, Japan; ^4^ Engineering Biology Research Center, Kobe University, Kobe, Japan; ^5^ Department of Food Science and Nutrition, Nagoya Women’s University, Nagoya, Japan; ^6^ Department of Environmental Health, Harvard University T.H Chan School of Public Health, Boston, MA, United States

**Keywords:** extracellular vesicle, skeletal muscle, macrophage, itaconate, IRG1

## Abstract

**Introduction:**

Macrophages play an important role in the innate immunity. While macrophage inflammation is necessary for biological defense, it must be appropriately controlled. Extracellular vesicles (EVs) are small vesicles released from all types of cells and play a central role in intercellular communication. Skeletal muscle has been suggested to release anti-inflammatory factors, but the effect of myotube-derived EVs on macrophages is unknown. As an anti-inflammatory mechanism of macrophages, the immune responsive gene 1 (IRG1)-itaconate pathway is essential. In this study, we show that skeletal muscle-derived EVs suppress macrophage inflammatory responses, upregulating the IRG1-itaconate pathway.

**Methods:**

C2C12 myoblasts were differentiated into myotubes and EVs were extracted by ultracentrifugation. Skeletal myotube-derived EVs were administered to mouse bone marrow-derived macrophages, then lipopolysaccharide (LPS) stimulation was performed and inflammatory cytokine expression was measured by RT-qPCR. Metabolite abundance in macrophages after addition of EVs was measured by CE/MS, and IRG1 expression was measured by RT-PCR. Furthermore, RNA-seq analysis was performed on macrophages after EV treatment.

**Results:**

EVs attenuated the expression of LPS-induced pro-inflammatory factors in macrophages. Itaconate abundance and IRG1 expression were significantly increased in the EV-treated group. RNA-seq analysis revealed activation of the PI3K-Akt and JAK-STAT pathways in macrophages after EV treatment. The most abundant miRNA in myotube EVs was miR-206-3p, followed by miR-378a-3p, miR-30d-5p, and miR-21a-5p.

**Discussion:**

Skeletal myotube EVs are supposed to increase the production of itaconate *via* upregulation of IRG1 expression and exhibited an anti-inflammatory effect in macrophages. This anti-inflammatory effect was suggested to involve the PI3K-Akt and JAK-STAT pathways. The miRNA profiles within EVs implied that miR-206-3p, miR-378a-3p, miR-30d-5p, and miR-21a-5p may be responsible for the anti-inflammatory effects of the EVs. In summary, in this study we showed that myotube-derived EVs prevent macrophage inflammatory responses by activating the IRG1-itaconate pathway.

## Graphical Abstract

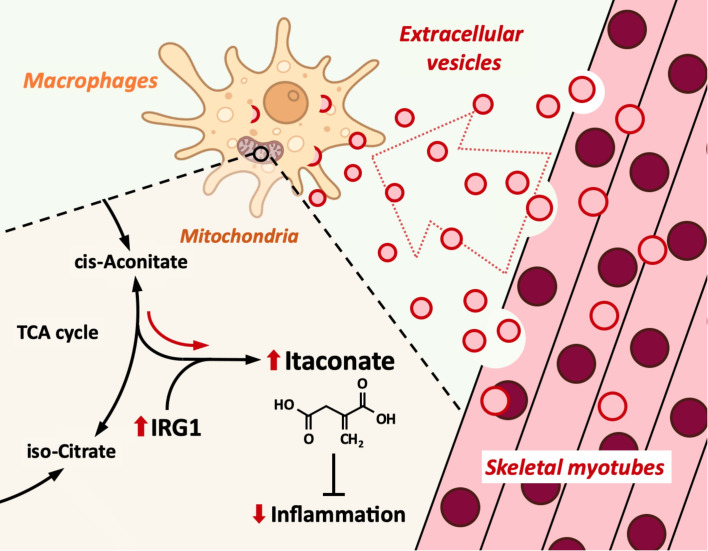

## Introduction

Macrophages play an important role in the innate immune system and represent the front line of defense against bacterial infections (1). They become activated in a pro-inflammatory way upon the detection of lipopolysaccharide (LPS), as characterized by the elevated expression of interleukin-1β (IL-1β), IL-6, and tumor necrosis factor-α (TNF-α) through the NF-κB pathway (2). During several inflammatory diseases, suppressing excessive inflammatory responses of macrophages is crucial to avoid tissue damage (3).

Recently, mesenchymal stem cell-derived extracellular vesicles (EVs) have been reported to have an anti-inflammatory effect on macrophages (4). EVs are lipid bilayer vesicles released from all types of cells and play a pivotal role in intercellular communication by encapsulating and delivering mRNAs, miRNAs, proteins, cytokines, and nucleic acids to distant organs and cells (5). Also, cells change the release kinetics of EVs in response to various stimuli, and the contents in EVs also change in response to the cellular microenvironment (6).

Skeletal muscle is usually recognized as a locomotory organ, on the other hand, it is also known as the largest secretory organ in the human body and involved in as much as 75% of the total metabolism in the body (7). Furthermore, skeletal muscle is the only secretory organ that can be stimulated noninvasively and readily because it is widely distributed on the surface of the body and is a voluntarily controllable organ. In fact, it has been reported that high-intensity exercise with muscle contraction increases circulating EV amount (8) and that high-intensity ultrasound stimulation to cultured myotubes promotes EV secretion (9). Thus, secretion of skeletal muscle-derived EVs can be more easily controlled than that of other organ-derived EVs, so the effect of skeletal muscle-derived EVs on macrophages is the key to controlling systemic inflammation. Furthermore, skeletal muscle is reported to secrete anti-inflammatory/immune modulatory factors (10). However, the effect of skeletal myotube-derived EVs on macrophage inflammation has not been clarified yet.

As an important anti-inflammatory factor in macrophages, itaconate has been attracting attention. Itaconate, a tricarboxylic acid (TCA) cycle derivative, is known for its anti-inflammatory, antioxidant, anti-tumor, and anti-microbial properties (11). Immune-responsive gene 1 (IRG1) has been regarded as a gene coding for immune-responsive gene 1 protein/cis-aconitic acid decarboxylase, an enzyme that catalyzes the production of itaconate by decarboxylating cis-aconitate (12). During infection, macrophages reprogram immunometabolism by increasing itaconate production *via* upregulating IRG1 expression (13). It is reported that for IRG1 activation, activities of the GR and JAK/STAT signaling pathways and the transcription factors C/ebpβ and Stat3 are required (14).

Here we show that skeletal myotube-derived EVs suppress macrophage inflammatory responses by inducing itaconate production in macrophages *via* upregulation of IRG1 expression.

## Materials and methods

### Cell culture

C2C12 myoblasts, mouse skeletal muscle cells, were purchased from ATCC. Myoblasts were seeded and cultured in 10 cm tissue culture dishes under 5% CO_2_ in Dulbecco’s modified Eagle’s medium (DMEM) supplemented with 10% fetal bovine serum (FBS). When the cells reached 90% confluence, the growth medium was changed to a differentiation medium (DMEM supplemented with 2% horse serum) and differentiation into myotubes was started. After differentiation for 6 days refreshing the medium every 48 h, the efficiency of differentiation was confirmed by observing contraction by electrical stimulation (**Supplemental Material 1**). After differentiation, EVs were collected by incubating the myotubes in serum-free DMEM for 6 h.

To obtain bone marrow-derived macrophages (BMDMs), bone marrow cells were harvested from femurs and tibias of 7-week-old male C57BL/6J mice and cultured in a Petri dish under 5% CO_2_ for eight days in RPMI 1640 with 10% FBS, 25% L929 cell supernatant, 1% Penicillin/Streptomycin, and 1% L-Glutamine. Differentiated BMDMs were plated in a 12-well tissue culture plate at a density of 3.0×10^5^/well with macrophage culture media (RPMI 1640 supplemented with 10% FBS, 10% L929 cell supernatant, 1% Penicillin/Streptomycin, and 1% L-Glutamine).

The present study was approved by the Institutional Animal Care and Use Committee and all experiments were performed according to the Kobe University Animal Experimentation Regulations.

### EV extraction and addition to BMDMs

Myotube-derived EVs were isolated by ultracentrifugation following a previously described method (15). Briefly, collecting medium was centrifuged at 1,000g for 10 min, followed by a second spin at 10,000g for 30 min to remove cell debris. Supernatant was collected and filtered through a 0.22 μm membrane, followed by a final centrifugation at 100,000g for 2 h to pellet EVs. The pellet was resuspended in macrophage culture media and filtered through a 0.22 μm membrane. Then, EVs were added to BMDMs at the concentration of 5.0×10^3^ particles/cell. After 1.5 h-treatment by EVs, the cells were incubated with culture media overnight without myotube-derived EVs.

### EV characterization by tunable resistive pulse sensing, Western blotting and flow cytometry

The isolated EVs were characterized by their size and the presence of the EV marker CD63 (16). The size distribution and concentration of EVs were measured using tunable resistive pulse sensing by qNano (Izon). The positive rate of CD63 in the collected EVs was analyzed using flow cytometry with a fluorescence-labeled CD63 antibody (Bio Legend Ltd., Japan) and magnetic beads coupled to a phosphatidylserine (PS)-binding protein (PS CaptureTM Exosome Flow Cytometry Kit, Fujifilm Wako Pure Chemical Co.) following the manufacturer’s instruction. EV-bound beads positive for CD63 antibody or isotype control were counted, and the positive rates were calculated using CytExpert (Beckman Coulter) software. Searching for the population of EV-bound beads using forward scatter (FSC) and side scatter (SSC) plots, the exosome population without aggregation was gated and the fluorescent signal in the corresponding histogram was evaluated. For Western blotting, EV proteins were extracted with 2% SDS sample buffer for 10 min at 80°C and were migrated on 12.5% sodium dodecyl sulfate-polyacrylamide gel. Following electrophoresis, proteins were transferred to a polyvinylidene difluoride membrane. The membrane was blocked with 5% skim milk for 10 min at room temperature and then immunoblotted with anti-CD63 (1:200 dilution, sc-5275, Santa Cruz) at 4°C overnight. The membrane was then incubated with horseradish peroxidase-conjugated secondary antibodies (1:10,000, GE Healthcare, Waukesha, WI) for an hour. The membrane was detected using EzWestLumi One (ATTO) enhanced chemiluminescence solution. Finally, images were captured using the LAS-1000 imaging system (Fujifilm) with a chemiluminescent image analyzer.

### Cell viability assessment by Zombie Red™ immunofluorescence staining

The viability of BMDMs was analyzed 24 h after EV treatment or treatment with 1% povidone-iodine (positive control) using Zombie Red™ as previously described (9). Briefly, the cells were washed twice with PBS and stained with Zombie Red™ solution (1:1000) for 15 min. Then, BMDMs were fixed using 4% paraformaldehyde for 30 min. After fixation, nuclei were stained with DAPI (1μg/mL) for 5 min. Stained images were observed using a BX50 fluorescence microscope at ×200 magnification (Olympus, Tokyo, Japan) and recorded with a digital camera (EOS Kiss X4, Canon, Tokyo, Japan). The numbers of total cells (blue) and dead cells (red) were counted and the percentage of live cells to total cells was calculated. Triplicate cell cultures and 5 random fields of each well were analyzed for each condition.

### RT-qPCR analysis

mRNAs from macrophages were isolated by TRIzol RNA Isolation protocol and used to make cDNA with iScriptTM cDNA Synthesis Kit (Bio-Rad). A StepOne™ Real-Time PCR thermal cycler was used to analyze the samples under the following conditions: 95° (3 min), 40 cycles of 95° (10 sec), and 60° (30 sec). The reaction mixture consisted of 8 μL cDNA, 1.5 μL 10× buffer, 0.3 μL 10 mM dNTPs, 1.5 μL 5 μM primers for each gene used in the study (F+R), 3.58 μL H_2_O, 0.075 μL Go Taq DNA polymerase, and 0.045 μL 2× SYBR green (Invitrogen). Target genes were *Il-1β*, Tnf-α, *Il-6*, *Nf-kB p65*, *Nf-kB p50*, and *Irg1*. For the analysis of pro-inflammatory genes, the cells were stimulated with 100 ng/mL LPS for 1.5 h at 24 h after EV treatment. Relative expression values for target genes were calculated by normalization to the expression of Glyceraldehyde-3-phosphate dehydrogenase (GAPDH). Obtained data were analyzed by the delta/delta CT method (17). The results are expressed as relative values with the control group or LPS-unstimulated group. The sequences for RT-qPCR primers are shown in **Supplemental Material 2**. Quadruplicate cell cultures and technical duplicates for each sample were analyzed.

### Metabolite analysis

At 24 h after EV treatment, BMDMs were washed with PBS twice and lysed in 80% methanol containing 50 μM (+)-10-camphorsulfonic acid, 400 μM L-methionine sulfone, and 400 μM piperazine-1,4-bis(2-ethanesulfonic acid) (PIPES) as internal standards. The cells were incubated for 15 min at -80°, then scraped and centrifuged at 14,000 g for 5 min at 4°. The supernatant was collected and filtered using a Millipore 5 kDa cut-off membrane to remove solubilized proteins. The dried metabolites were dissolved in Milli-Q water after evaporation of the aqueous-layer extracts under vacuum using a FreeZone 2.5 Plus freeze dry system (Labconco, Kansas City, MO). The concentrations of intracellular metabolites were analyzed with a CE/MS (CE, Agilent G7100; MS, Agilent G6224AA LC/MSD TOF; Agilent Technologies, Palo Alto, CA) controlled by MassHunter Workstation Data Acquisition software (Agilent Technologies), as described previously (18). The same validation was performed on BMDMs stimulated with LPS for 1.5 h at 24 h after EV treatment. For metabolite analysis on myotube-derived EVs, EVs extracted as above were used. The relative abundance of each metabolite to the control group or LPS group was calculated. Quadruplicate cell cultures were analyzed for each condition tested.

### RNA sequencing of BMDMs and myotube-derived EVs

Total RNA was extracted from BMDMs using TRIzol reagent (Takara Biotechnology, Japan) according to the manufacturer’s instructions. Raw RNA sequence data were obtained using an Illumina NovaSeq™ 6000 machine. After acquiring the raw data, the fold change (mean of each RNA in the EV group/mean of each RNA in the control group) and P-values were calculated for each RNA. These P-values were used to calculate the false discovery rate (FDR) for each RNA, which was further used as a filter to identify significant RNAs with a fold change ≥ 2 or ≤ 0.5 and an FDR < 0.05. The R 3.5.3 program was used to create the volcanic plots. The 20 most enriched pathways related to signaling transduction are presented and were used to reveal the associated pathways after a pathway analysis with the Kyoto Encyclopedia of Genes and Genomes (KEGG) pathway database. For miRNA analysis in myotube-derived EVs, miRNA was extracted from isolated EVs and used to characterize miRNA profile in skeletal myotube-derived EVs using the above-mentioned method.

### Statistical analysis

All values are presented as mean ± SD. Statistical analysis was performed with Statistical 4 (OMS, Tokyo, Japan). Student’s t-test was used for two-group comparisons, and ANOVA (with Tukey’s multiple comparison test as a *post-hoc* analysis) was used for multiple comparisons. The sample size for each test needed to generate a power of at least 0.8 at a significance level of 0.05 (α = 0.05, β = 0.2) was calculated using power analysis using G Power software (19).

## Results

### Characterization of myotube-derived EVs

**Figure 1:**
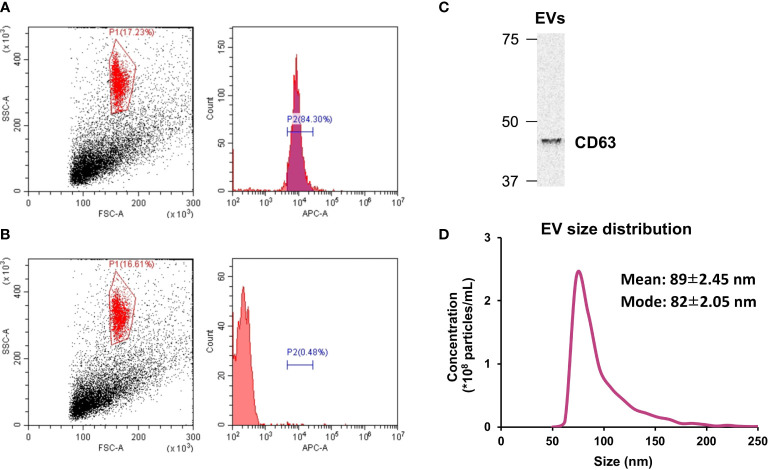
Extracellular vesicle **(EV)** characterization. **(A)** The presence of CD63 on isolated EVs was verified by flow cytometry. **(B)** CD63 was absent in particles treated with the isotype control. **(C)** Western blot analysis of CD63. **(D)** Size distribution of isolated EVs was measured by tunable resistive pulse sensing. n = 3.

Isolated EVs were characterized using flow cytometry, Western blotting, and tunable resistive pulse sensing. As shown in **Figure 1A**, 84.3% of the isolated particles were found to be positive for CD63 while less than 1% of the particles treated with the isotype control showed the presence of CD63 (**Figure 1B**). In addition, presence of CD63 in the extracted EVs was also confirmed by Western blotting (**Figure 1C**). Regarding the size of the extracted EVs, most particles were within the size of 50-200 nm, which is EV size range (**Figure 1D**) (20).

### Myotube-derived EVs attenuate LPS-induced inflammatory responses in BMDMs

**Figure 2:**
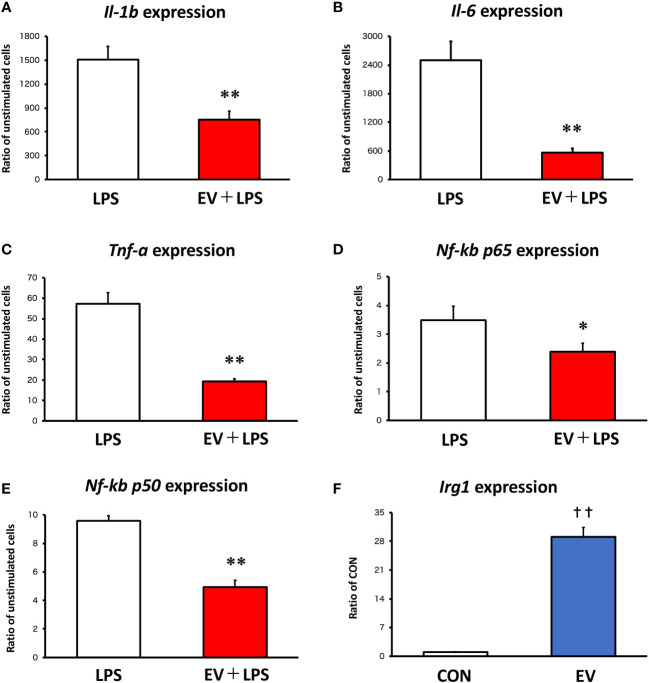
mRNA expression levels in macrophages by qPCR. **(A-E)** Bone marrow-derived macrophages were stimulated with 100 ng/mL lipopolysaccharide (LPS) 24 h after extracellular vesicle **(EV)** treatment and the expression levels of pro-inflammatory genes were measured. **(F)** *Irg1* expression level in macrophages was measured after EV treatment. *p < 0.05, **p < 0.01 compared with LPS, **p < 0.01 compared with LPS, ^††^ *p* < 0.01 compared with CON (Student’s *t*-test). n = 4. Mean ± SD shown.

Twenty-four hours after EV treatment, BMDMs were treated with 100 ng/mL LPS for 1.5 h as an inflammation model. To assess the effect of myotube-derived EVs on macrophage inflammation, mRNA expression levels of pro-inflammatory *Il-1β*, *Il-6*, and *Tnf-α* were measured. As shown in **Figures 2A-E**, LPS significantly upregulated the expression levels of *Il-1β*, *Il-6*, *Tnf-α, Nf-kb p65*, and *Nf-kb p50* in BMDMs and myotube-derived EVs significantly prevented the upregulation of those factors. Furthermore, to investigate the effect of myotube-derived EVs on IRG1 expression in BMDMs, mRNA expression of *Irg1* in BMDMs after EV treatment was measured. As shown in **Figure 2F**, EV treatment significantly increased the mRNA expression of *Irg1* in BMDMs.

### Myotube-derived EVs have no cytotoxicity on BMDMs

**Figure 3:**
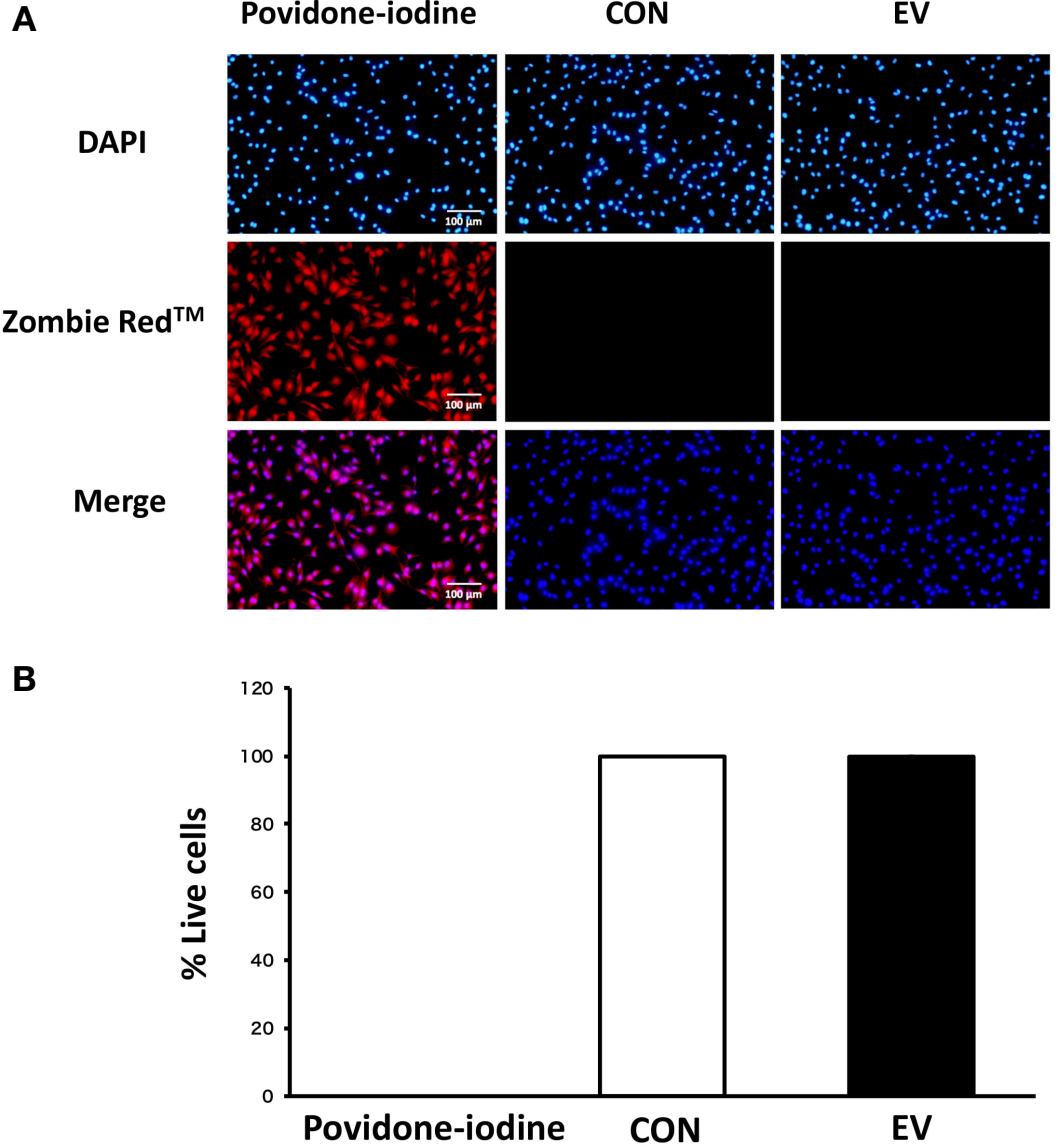
Cell viability 24 h after extracellular vesicle (EV) treatment. **(A)** Bone marrow-derived macrophages were stained with Zombie Red™ immunofluorescence reagent. After fixation, the cells were counter-stained with DAPI. **(B)** The percentage of live cells to total cells was calculated. Triplicate cell cultures were analyzed and 5 random fields of each well were examined. Mean ± SD shown.

The cell viability of BMDMs was measured using Zombie Red™ immunofluorescence staining 24 h after EV treatment in order to examine the cytotoxicity of myotube-derived EVs on macrophages. As shown in **Figure 3**, while cells treated with 1% povidone-iodine showed a significant decline in viability, EV treatment did not result in cell damage.

### Myotube-derived EVs induce itaconate production in BMDMs

**Figure 4:**
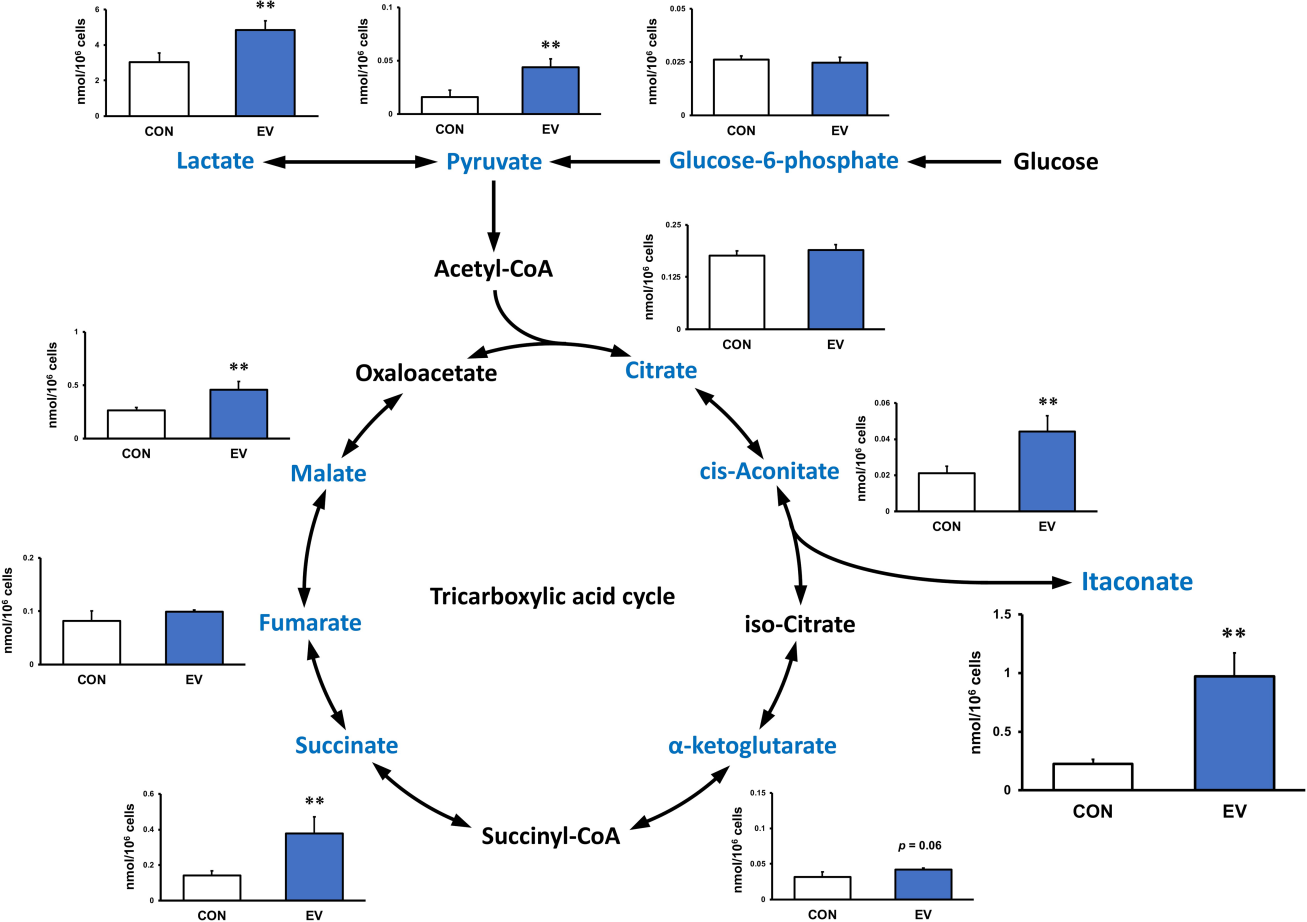
Metabolite profile in bone marrow-derived macrophages treated by myotube-derived extracellular vesicles. **p < 0.01 compared with CON (Student’s *t*-test). n = 4. Mean ± SD shown.

**Figure 5:**
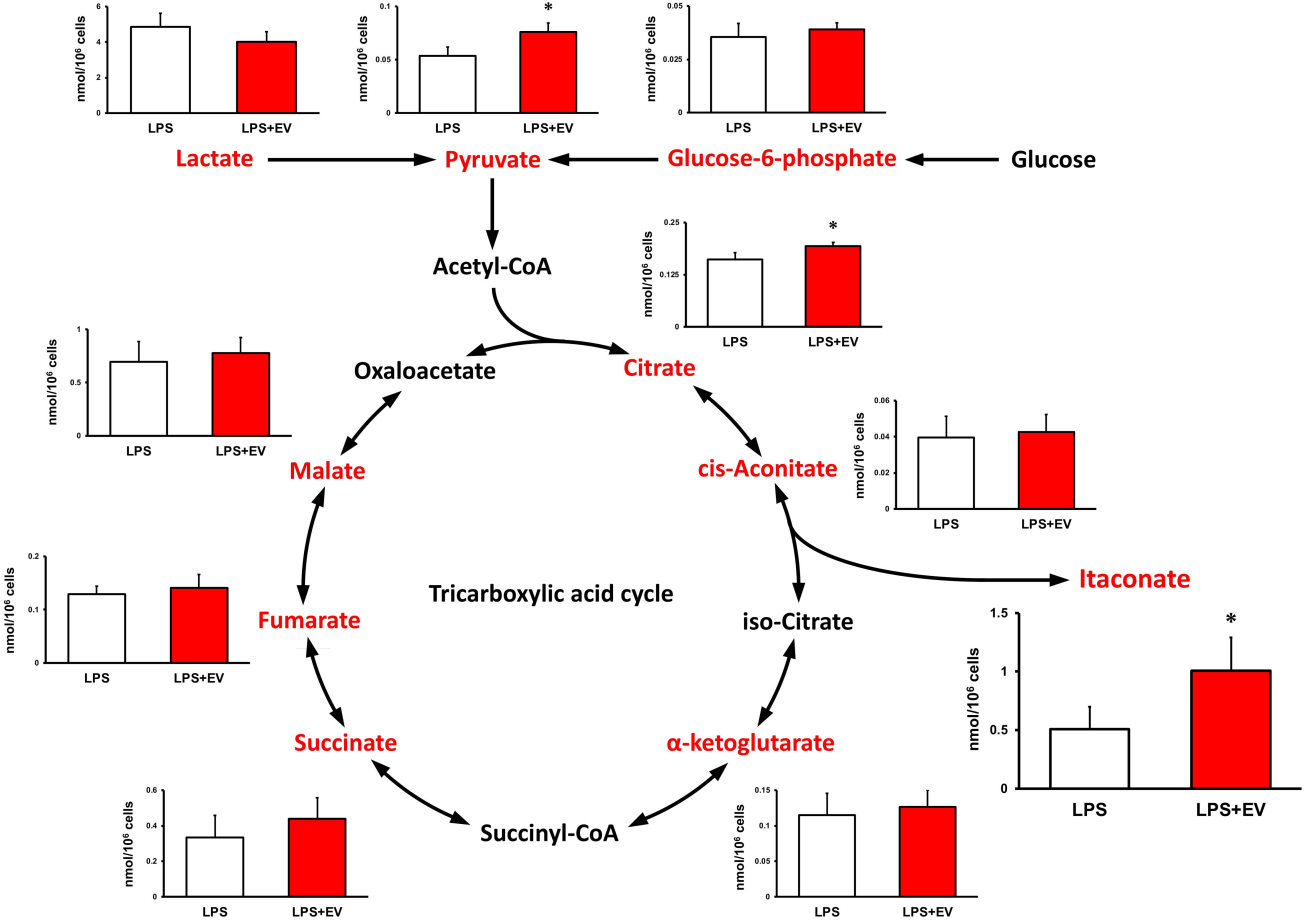
Metabolite profile in bone marrow-derived macrophages treated by myotube-derived extracellular vesicles. *p < 0.05 compared with LPS (Student’s *t*-test). n = 4. Mean ± SD shown.

Intracellular levels of the metabolites were measured 24 h after EV treatment to assess the effect of myotube-derived EVs on the metabolite profile in BMDMs. As shown in **Figure 4**, itaconate was the most significantly elevated of the metabolites measured. Moreover, EVs induced an overall increase in metabolites in the TCA cycle. Subsequently, to assess the effect of EV treatment on the metabolite profile in BMDMs upon LPS stimulation, BMDMs were stimulated with LPS for 1.5 h at 24 h after EV treatment and the intracellular levels of the metabolites were quantified. As a result, EV+LPS group showed a significantly higher level of itaconate compared to LPS group (**Figure 5**).

### Metabolite analysis in myotube-derived EVs

To investigate the metabolite profile in C2C12 myotube-derived EVs, the abundance of metabolites in extracted EVs was quantified. As shown in **Table 1**, nine metabolites were detected and myotube-derived EVs were rich in lactate and pyruvate.

**Table 1:** Metabolite profile in myotube-derived EVs by CE/MS.

Metabolite	Abundance (nmol/10^10^ particles)
Lactate	153.499
Pyruvate	5.042
Citrate	0.247
Succinate	0.156
α-ketoglutarate	0.055
Malate	0.052
cis-Aconitate	0.028
Itaconate	0.009
D-Glucose 6-phosphate	0.007

### RNA-seq analysis of EV-treated BMDMs

**Figure 6:**
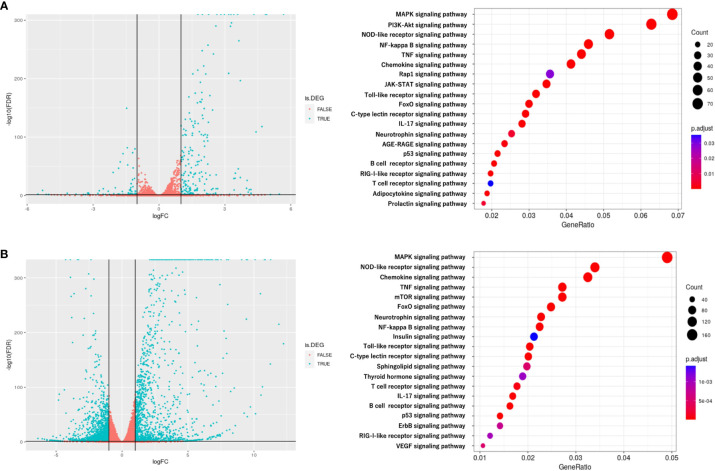
RNA sequencing analysis of BMDMs after extracellular vesicle (EV) treatment. **(A)** Left: Volcano plot of differentially expressed RNAs in control group vs. EV group. Blue dots represent RNAs with statistically significant difference and red dots show RNAs with no statistically significant difference between EV group vs. control group. Right: Kyoto Encyclopedia of Genes and Genomes (KEGG) analysis on differentially expressed RNAs; the 20 most enriched pathways linked to signaling transduction are presented. **(B)** Left: Volcano plot of differentially expressed RNAs in control group vs. LPS group. Blue dots represent RNAs with statistically significant difference and red dots show RNAs with no statistically significant difference between control group vs. LPS group. Right: Kyoto Encyclopedia of Genes and Genomes (KEGG) analysis on differentially expressed RNAs; the 20 most enriched pathways linked to signaling transduction are presented. n = 3.

To investigate the mechanism by which myotube-derived EVs activated the IRG1-itaconate pathway in BMDMs, RNA sequencing analysis of BMDMs after EV treatment was performed. A total of 14,784 RNAs were identified by proteomic quantitative analysis, and according to the standard of a fold change of ≥ 2 or ≤ 0.5 as well as an FDR < 0.05, we screened 268 up-regulated RNAs and 95 down-regulated RNAs in the EV group versus the control group (**Supplemental Material 3**). Differentially expressed RNAs are displayed as a volcano plot (**Figure 6A**) and the top 10 of upregulated RNAs are shown in **Table 2**. Cxcl1/2 were the most upregulated RNAs in BMDMs by myotube-derived EV treatment. Enrichment pathway analysis was also conducted to identify the most activated pathways linked to signaling transduction after EV treatment. The 20 most enriched pathways are shown in **Figure 6A**, which included the PI3K-Akt, JAK-STAT, and adipocytokine signaling pathways. To compare the physiological action between myotube-derived EVs and pathological endotoxin, the same analyses were performed on BMDMs stimulated by LPS alone. Differentially expressed RNAs by LPS treatment are displayed as a volcano plot and the 20 most enriched pathways are shown in **Figure 6B**. The top 10 of upregulated RNAs by LPS stimulation are shown in **Table 3**. As a result, in LPS-stimulated group, Il12b was the most upregulated gene and the PI3K-Akt, JAK-STAT, and adipocytokine signaling pathways were not in the 20 most enriched pathways unlike the EV-treated group.

**Table 2:** Top 10 of upregulated RNAs in macrophages after extracellular vesicle (EV) treatment. Fold change and P-value of EV group vs. control group are shown. Mean ± SD shown.

Gene	Fold change	P-value
Cxcl1	49.6 ± 2.19	< 0.001
Cxcl2	27.8 ± 1.45	< 0.001
Il1b	25.1 ± 3.34	< 0.001
Mir155hg	20.8 ± 1.75	< 0.001
Gm17041	19.7 ± 2.62	< 0.001
Sox7	19.0 ± 5.10	< 0.01
Tpbg	18.7 ± 0.94	< 0.001
Pgf	16.2 ± 0.57	< 0.001
Vcam1	16.2 ± 4.40	< 0.001
Cish	12.8 ± 1.66	< 0.001

**Table 3:** Top 10 of upregulated RNAs in macrophages after LPS treatment. Fold change and P-value of EV group vs. control group are shown. Mean ± SD shown.

Gene	Fold change	P-value
Il12b	2517 ± 267	< 0.001
Plat	1869 ± 184	< 0.001
Il1a	1452 ± 145	< 0.001
Il1b	1508 ± 154	< 0.001
Mir155hg	1029 ± 174	< 0.001
Cxcl3	620 ± 117	< 0.001
Adamts4	713 ± 90.6	< 0.001
Serpine1	631 ± 66.3	< 0.001
Cxcl1	557 ± 54.3	< 0.001
Ptgs2	466 ± 26.2	< 0.001

### miRNA profile in myotube-derived EVs

**Figure 7:**
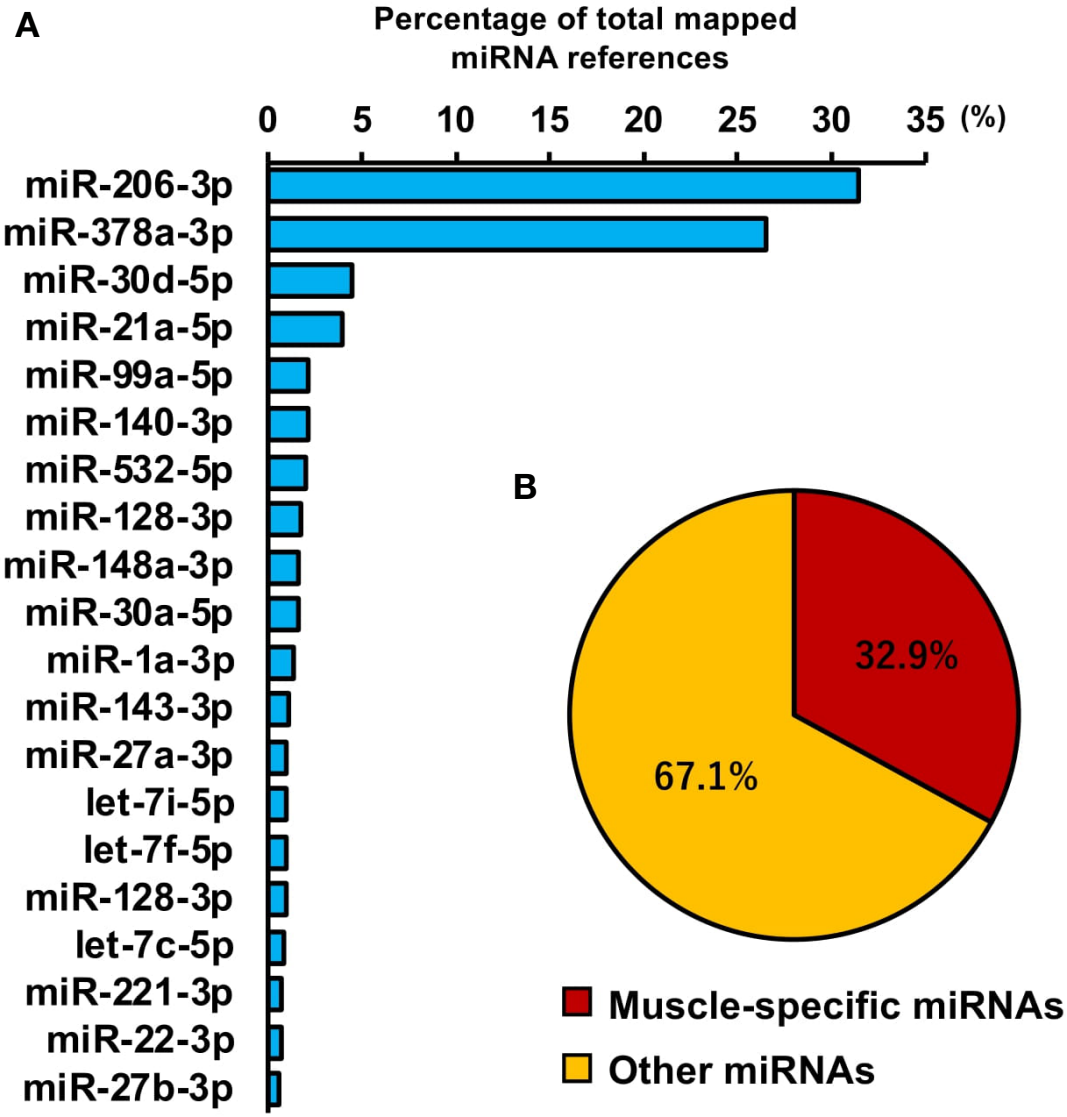
miRNA profile in skeletal myotube-derived extracellular vesicles (EVs). miRNA-sequencing analysis was performed on miRNAs extracted from the EVs. **(A)** The 20 most abundant miRNAs in myotube EVs. **(B)** Ratio of muscle-specific miRNAs to total miRNAs. n = 2.

To investigate miRNAs contained in EVs, miRNA-seq analysis was performed. A total of 442 miRNAs were identified by proteomic quantitative analysis (**Supplemental Material 4**). The 20 most enriched miRNAs in the EVs are shown in **Figure 7A** and miR-206-3p was the most abundant miRNA, followed by miR-378a-3p, miR-30d-5p, and miR-21a-5p. Muscle-specific myomiRNAs accounted for 32.9 percent of total mapped miRNAs (**Figure 7B**).

### Macrophage response to myotube-derived EV treatment in inflammation-related genes

**Figure 8:**
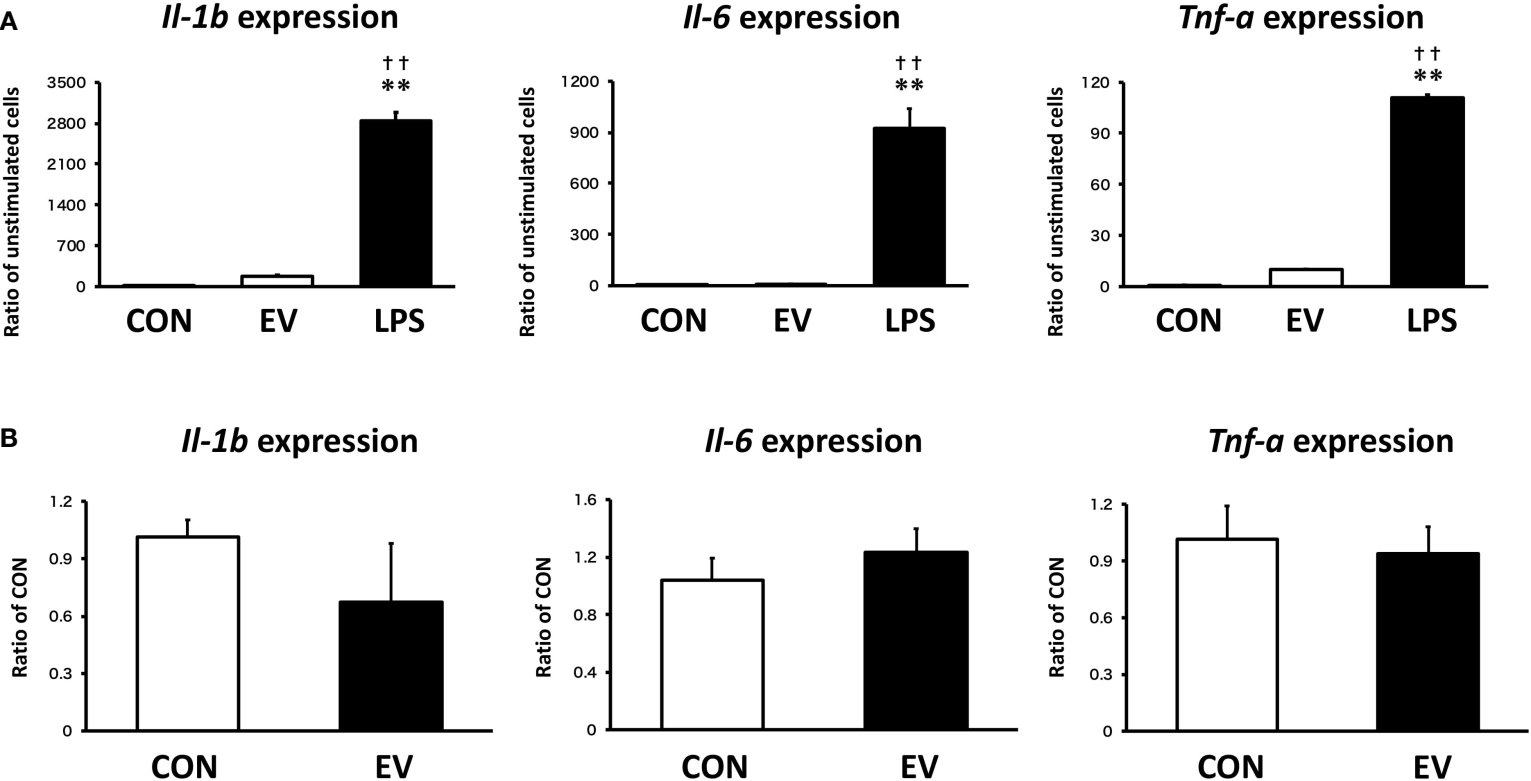
Macrophage responses to myotube-derived extracellular vesicle **(EV)** treatment in inflammation-related genes. **(A)** mRNA expression levels of pro-inflammatory factors were measured by qPCR after 1.5 h-treatment by skeletal muscle EVs or LPS. **p < 0.01 compared with CON, ^††^ *p* < 0.01 compared with LPS (Tukey’s multiple comparison test). **(B)** mRNA expression levels of pro-inflammatory factors were measured by qPCR 24 h after EV treatment. n = 4. Mean ± SD shown.

To assess the response of macrophages to myotube EVs, pro-inflammatory mRNA expression levels in macrophages after EV treatment were measured. As shown in **Figure 8A**, EVs slightly upregulated the expression levels of pro-inflammatory factors in macrophages, but the response was very minor compared to the elevation caused by LPS. Furthermore, the expression levels of those pro-inflammatory factors were almost the same level as the control group 24 h after the treatment (**Figure 8B**).

## Discussion

This is the first report showing that skeletal myotube-derived EVs have an anti-inflammatory effect on macrophages. Skeletal myotube EVs prevented LPS-induced overexpression of inflammatory factors *Il-1b*, *Il-6*, and *Tnf-a* and suppressed the upregulation of the expression of *Nf-kb* in macrophages without causing a reduction in cell viability.

A variety of illnesses occur and develop as a result of the overexpression of IL-1β, IL-6, and TNF-α. Numerous inflammatory disorders have been found to improve with the suppression of these pro-inflammatory factors, and experiments evaluating their blockade have also been carried out (21). Thus, the modulation of pro-inflammatory factors by myotube-derived EVs raises the possibility of a novel strategy for immune regulation utilizing skeletal muscle, which is the largest and most approachable secretory organ.

As the mechanism by which myotube-derived EVs exerted an anti-inflammatory effect in macrophages, *Irg1* expression was upregulated in EV-treated macrophages, followed by an increase in the level of itaconate. Additionally, after LPS stimulation, EV-treated macrophages retained a higher level of itaconate compared to the untreated group. Given that IRG1 catalyzes the synthesis of itaconate (12), it is anticipated that myotube-derived EVs enhanced itaconate production in macrophages *via* IRG1 upregulation. Itaconate has been shown to reduce the severity of a variety of inflammatory disorders by reducing macrophage inflammatory responses (22–25). Furthermore, Lampropoulou et al. reported that itaconate alleviates inflammatory responses of macrophages in a concentration-dependent manner (26). Therefore, enhancing itaconate synthesis in macrophages is crucial for the management of inflammatory disorders.

In this study, skeletal myotube-derived EVs most upregulated Cxcl1 and Cxcl2 expression levels in macrophages. Cxcl1 and Cxcl2 have been reported to activate the IRG1-inducing factor Protein Kinase C (PKC) (27–29). Whereas, during LPS-induced inflammation, other factors were preferentially upregulated. This suggests that skeletal myotube EVs trigger different responses in macrophages than the typical inflammatory responses induced by endotoxin. In addition, the result of pathway enrichment analysis shows that the PI3K-Akt signaling pathway, which induces PKC, the IL-17 signaling pathway, which induce C/ebpβ, the adipocytokine signaling pathway, which induces STAT3, and the JAK-STAT signaling pathways were within the top 20 most enriched pathways in EV-treated macrophages. Hall et al. reported that C/ebpβ, STAT3, and JAK-STAT pathway are involved in IRG1 activation (14). On the other hand, in the top 20 most enriched pathways activated during LPS-induced inflammation, the PI3K-Akt, JAK-STAT, and adipocytokine signaling pathways, which were elevated after EV treatment, were not included. Thus, it is assumed that skeletal myotube-derived EVs activated the IRG1-itaconate pathway *via* multiple pathways, eliciting a response distinct from endotoxin-induced inflammatory responses.

miRNA analysis revealed that miRNA profile in skeletal muscle EVs is largely composed of miR-206-3p, a skeletal muscle-specific myomiRNA (30), and miR-378a-3p, a muscle-enriched miRNA (31). Lin et al. reported that transfection of miR-mimic-206-3p into macrophages suppressed macrophage inflammation and transfection of miR-inhibitor-206-3p increased the level of inflammatory factors in macrophages (32). Rückerl et al. identified miR-378a-3p as a factor contributing to the induction of anti-inflammatory macrophage reprogramming (33). In addition, Kris et al. reported that miR-378a has anti-inflammatory effects on macrophages and its deficiency enhances severity of inflammation (34). Taken together, miR-206 and miR-378a, which were abundant in skeletal myotube EVs, have been reported to exert anti-inflammatory effects in macrophages, but their detailed mechanisms and effects on the activation of the IRG1-itaconate pathway are still unclear and further studies are expected. Meanwhile, miR-30d, which was the third most abundant miRNA in skeletal myotube EVs, is reported to activate the JAK-STAT pathway by suppression of SOCS1 and SOCS3, negative regulators of the JAK-STAT pathway (35, 36). Furthermore, the fourth most abundant miR-21a is reported to target PI3K-Akt inhibitor PTEN and downregulate its expression (37). Based on these, it is suggested that these miRNAs may be involved in the activation of the JAK-STAT pathway and PI3K-Akt pathway in macrophages by skeletal myotube-derived EVs.

In this study, skeletal myotube EVs caused an elevation of inflammatory factors in macrophages to some extent. However, this response was dramatically small compared to the LPS-induced elevation of those factors. Moreover, after 24 h of EV treatment, the level of inflammatory factors recovered to the same level as non-treated group, suggesting that the inflammatory response caused by skeletal myotube-derived EVs is not a pathological hyperinflammatory response but a natural process of immunometabolism.

Additionally, myotube-derived EVs promoted the metabolism of the TCA cycle in macrophages as well as upregulation of lactate and pyruvate. Myotube EVs also increased the gene expression of phosphofructokinase, hexokinase, and pyruvate kinase, the rate-limiting enzymes of glycolysis, and isocitrate dehydrogenase and 2-oxoglutarate dehydrogenase, the rate-limiting enzymes of the TCA cycle (**Supplemental Material 5**), indicating that the EVs enhanced the metabolism of both glycolysis and the TCA cycle in macrophages. Metabolite analysis on myotube-derived EVs revealed that they are rich in lactate and pyruvate. Hui et al. reported that lactate and pyruvate can be a major carbon source, and thus energy source, for the TCA cycle (38). Therefore, EVs possibly activated the TCA cycle by delivering myotube-derived lactate and pyruvate to macrophages. While the TCA cycle serves as an important regulatory function in driving energy production during macrophage activation, the accumulation of specific TCA cycle metabolites supports specific macrophage effector functions (39). Thus, skeletal myotube-derived EVs, which can increase metabolites in the TCA cycle without causing excessive inflammatory responses, may be utilized to control macrophage dynamics.

While this study showed that skeletal myotube-derived EVs exert an anti-inflammatory effect by activating the IRG1-itaconate pathway *via* multiple pathways in macrophages, the details of how each pathway is involved in the activation of IRG1 are still unclear. Also, validation of the pathways activated by EVs and identification of contents of the skeletal myotube EVs which were responsible for these effects are expected as further studies.

In summary, this study found that skeletal myotube-derived EVs prevent macrophage inflammatory responses by activating the IRG1-itaconate pathway. These findings suggest a new immunoregulatory strategy utilizing skeletal muscle-derived EVs.

## Data availability statement

The original contributions presented in the study are publicly available. These data can be found here: https://ngdc.cncb.ac.cn/omix. (OMIX repository, accession numbers OMIX003091 and OMIX003092).

## Ethics statement

The animal study was reviewed and approved by Kobe University Animal Care and Use Committee.

## Author contributions

AY: Conceptualization, Data curation, Formal analysis, Investigation, Methodology, Project administration, Validation, Visualization, Writing - original draft; NM: Conceptualization, Data curation, Formal analysis, Funding acquisition, Investigation, Methodology, Project administration, Resources, Software, Supervision, Validation, Visualization, Writing - original draft, Writing - review & editing; JY: Conceptualization, Supervision, Writing - review & editing; XM; Formal analysis, Investigation, Methodology, Validation, Visualization, Writing - original draft; MU: Formal analysis, Investigation, Methodology, Supervision, Validation, Visualization, Writing - original draft, Writing - review & editing; MM: Data curation, Formal analysis, Investigation, Software, Writing - review & editing; YN: Data curation, Funding acquisition, Resources, Software, Writing - review & editing; TH: Data curation, Formal analysis, Funding acquisition, Project administration, Resources, Software, Supervision, Writing - review & editing; HK: Conceptualization, Funding acquisition, Project administration, Supervision; HF: Conceptualization, Funding acquisition, Project administration, Resources, Supervision, Writing - review & editing; Z-MY: Conceptualization, Methodology, Project administration, Supervision, Writing - review & editing. All authors contributed to the article and approved the submitted version.
