# Supplementary material for: Skeletal myotube-derived extracellular vesicles enhance itaconate production and attenuate inflammatory responses of macrophages
Source: Front Immunol. 2023 Mar 2;14:1099799. doi: 10.3389/fimmu.2023.1099799 (PMC10018131; doi:10.3389/fimmu.2023.1099799)
Supplement: Supplementary file 1 [file DataSheet_1.zip › Supplemental material 1.PPTX]

## Slide 1
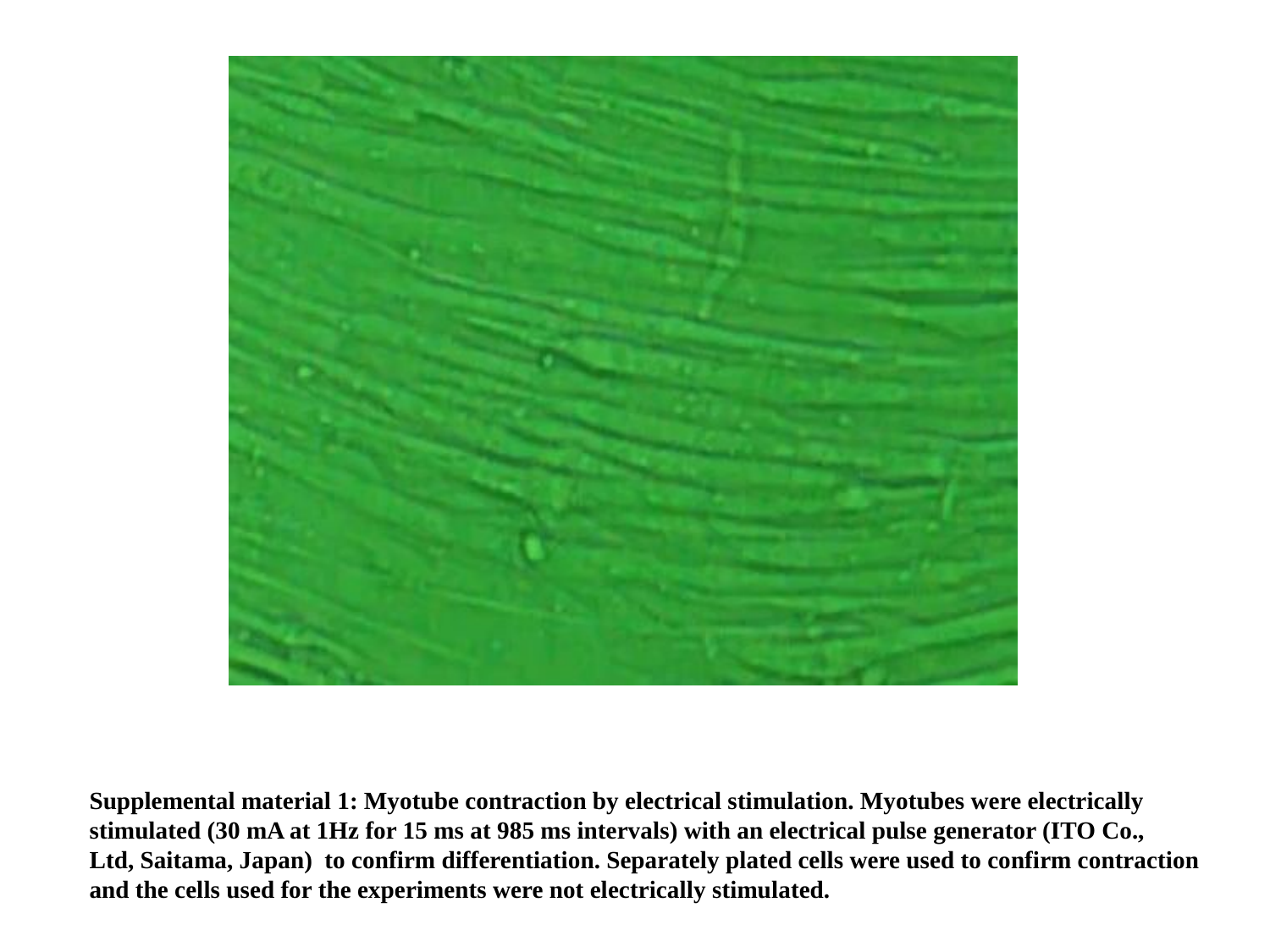

Supplemental material 1: Myotube contraction by electrical stimulation. Myotubes were electrically
stimulated (30 mA at 1Hz for 15 ms at 985 ms intervals) with an electrical pulse generator (ITO Co.,
Ltd, Saitama, Japan) to confirm differentiation. Separately plated cells were used to confirm contraction
and the cells used for the experiments were not electrically stimulated.
