# Supplementary material for: Skeletal myotube-derived extracellular vesicles enhance itaconate production and attenuate inflammatory responses of macrophages
Source: Front Immunol. 2023 Mar 2;14:1099799. doi: 10.3389/fimmu.2023.1099799 (PMC10018131; doi:10.3389/fimmu.2023.1099799)
Supplement: Supplementary file 1 [file DataSheet_1.zip › Supplemental material 2.DOCX]

**Supplemental material 2: Primer sequences for RT-qPCR**

| Primer | Sequence |
| --- | --- |
| GAPDH - Forward | 5'-CCAATGTGTCCGTCGTGGATCT-3' |
| GAPDH - Reverse | 5'-GTTGAAGTCGCAGGAGACAACC-3' |
| IL-1β - Forward | 5'-GCCTTGGGCCTCAAAGGAAAGAA-3' |
| IL-1β - Reverse | 5’-ATTGCTTGGGATCCACACTCTCC-3' |
| IL-6 - Forward | 5'-ACAAAGCCAGAGTCCTTCAGAGAG-3' |
| IL-6 - Reverse | 5'-TTGGATGGTCTTGGTCCTTAGCCA-3' |
| TNF-α - Forward | 5'-CCTATGTCTCAGCCTCTTCT-3' |
| TNF-α - Reverse | 5'-GGGAACTTCTCATCCCTTTG-3' |
| NFkB p65-Forward | 5'-CTCAACTTCTGTCCCCAAGCC-3' |
| NFkB p65-Reverse | 5'-GTTTGAGATCTGCCCTGATGG-3' |
| NFkB p50-Forward | 5'-GCACAGACGGTGTCTAGCAA-3' |
| NFkB p50-Reverse | 5'-GCGGAGGGACAGCAGTAACA-3' |
| IRG1 - Forward | 5'-GCAACATGATGCTCAAGTCTG-3' |
| IRG1 - Reverse | 5'-TGCTCCTCCGAATGATACCA-3' |
