# Supplementary material for: Skeletal myotube-derived extracellular vesicles enhance itaconate production and attenuate inflammatory responses of macrophages
Source: Front Immunol. 2023 Mar 2;14:1099799. doi: 10.3389/fimmu.2023.1099799 (PMC10018131; doi:10.3389/fimmu.2023.1099799)
Supplement: Supplementary file 1 [file DataSheet_1.zip › Supplemental material 3.DOCX]

**Supplemental material 3a: Table of upregulated RNAs in macrophages by skeletal muscle EVs**

| Gene | Fold change | P-value |
| --- | --- | --- |
| Cxcl1 | 49.6 | 6.2364E-06 |
| Cxcl2 | 27.7745358 | 1.2734E-05 |
| Il1b | 25.0888889 | 0.00052711 |
| Mir155hg | 20.7906977 | 9.2299E-05 |
| Gm17041 | 19.6666667 | 0.00065705 |
| Sox7 | 19 | 0.00858092 |
| Tpbg | 18.6666667 | 3.6649E-05 |
| Pgf | 16.2222222 | 4.3356E-06 |
| Vcam1 | 16.1666667 | 0.00871491 |
| Cish | 12.8235294 | 0.00054593 |
| Ptgs2 | 12.0403587 | 1.8321E-05 |
| Il1a | 11.9393939 | 0.00147044 |
| Tnf | 11.5455834 | 3.4486E-05 |
| Gem | 10.9166667 | 0.00011895 |
| Tmem200b | 10.8571429 | 0.00882175 |
| Cxcl3 | 10.8095238 | 0.00074193 |
| mt-Tt | 10.5 | 0.01910366 |
| Olr1 | 10.3783784 | 0.00172594 |
| Pde4b | 10.2621359 | 7.5536E-06 |
| Efcab8 | 10 | 0.42112254 |
| Acod1 | 9.35686275 | 7.2266E-05 |
| Cdc42ep2 | 8.71747212 | 7.4491E-06 |
| Gm13450 | 8.5 | 0.25081536 |
| Cd83 | 7.62630792 | 1.9845E-05 |
| Gm15419 | 7 | 0.29842893 |
| Tnfaip3 | 6.80337212 | 3.2073E-06 |
| Art2a-ps | 6.23076923 | 0.00332188 |
| Itgb8 | 6 | 0.01613009 |
| Tnfaip2 | 5.99759411 | 6.4487E-05 |
| Mefv | 5.95652174 | 0.00790643 |
| Art3 | 5.89516129 | 0.0040066 |
| Ccl3 | 5.82237648 | 0.0001369 |
| Gm13889 | 5.8 | 0.08112177 |
| Gm7019 | 5.66666667 | 0.01144691 |
| Lcn2 | 5.5 | 0.0790837 |
| Gm14023 | 5.36 | 0.00366882 |
| Lox | 5.25 | 0.13369223 |
| Gm26885 | 5.23982869 | 1.2915E-05 |
| Foxs1 | 5.2 | 0.00495204 |
| Spry1 | 5.13043478 | 0.00315576 |
| A1cf | 5 | 0.13640642 |
| Lif | 5 | 0.02058368 |
| Mycl | 4.84090909 | 0.00235284 |
| Ccl4 | 4.82198953 | 0.00043142 |
| Gm14963 | 4.71428571 | 0.0079662 |
| Traf1 | 4.59803922 | 0.00047385 |
| Gm28513 | 4.50632911 | 8.3165E-05 |
| Cxcl10 | 4.50057715 | 0.00181431 |
| Jdp2 | 4.46756329 | 0.00144622 |
| Spic | 4.46153846 | 0.05333826 |
| Kdm6b | 4.39598997 | 2.5717E-05 |
| Mir6244 | 4.3373494 | 8.6462E-05 |
| 1500005C15Rik | 4.25 | 0.1308043 |
| Gm16175 | 4.23015873 | 0.00019555 |
| Btg2 | 4.14785992 | 7.2935E-06 |
| Cxcl11 | 4.11111111 | 0.00194197 |
| Kdm6bos | 4.09090909 | 0.00028585 |
| Adora2b | 4.08037383 | 0.00046352 |
| Dusp2 | 4.0397351 | 4.2764E-05 |
| Rgs16 | 4.02793296 | 0.00015541 |
| Nod2 | 4.00816327 | 5.3565E-05 |
| Gm13650 | 4 | 0.38923536 |
| D630036H23Rik | 4 | 0.15059012 |
| Nfkbie | 3.96392063 | 3.5006E-05 |
| Pnrc1 | 3.93737575 | 5.3982E-05 |
| Bcl3 | 3.79849498 | 0.00016776 |
| Ccrl2 | 3.7960199 | 0.00139249 |
| Tlr2 | 3.77629278 | 7.38E-05 |
| Dlg5 | 3.75 | 0.3130804 |
| Gpr84 | 3.74697098 | 0.00017759 |
| Icosl | 3.72873409 | 1.3507E-05 |
| Flrt3 | 3.7037037 | 0.00035122 |
| Gpr132 | 3.67583213 | 0.0002197 |
| RP23-110E20.5 | 3.6453018 | 0.00015223 |
| Gm35876 | 3.57142857 | 0.02876834 |
| Cacnb3 | 3.57142857 | 0.13850842 |
| Icam1 | 3.56979461 | 0.00014592 |
| Irf1 | 3.52355512 | 2.0148E-05 |
| Dusp8 | 3.5037037 | 0.0002425 |
| Adora2a | 3.5 | 0.07420148 |
| Gm17334 | 3.47107438 | 0.00020884 |
| Hspa1b | 3.46710526 | 0.00023052 |
| Maff | 3.45273632 | 5.2388E-05 |
| Dbpht2 | 3.42857143 | 0.0605258 |
| Arl5c | 3.40752351 | 4.5539E-05 |
| Mfsd6l | 3.4 | 0.02742575 |
| Tagap | 3.39018692 | 1.5773E-05 |
| Tal2 | 3.33333333 | 0.08279449 |
| Mmp9 | 3.33333333 | 0.06100976 |
| Csf1 | 3.32305056 | 5.5546E-05 |
| E230013L22Rik | 3.29551451 | 0.00018128 |
| Nlrp3 | 3.28733398 | 3.5955E-05 |
| Plek2 | 3.28571429 | 0.08641805 |
| 1700058P15Rik | 3.25806452 | 0.0003836 |
| Gm18445 | 3.25 | 0.01107215 |
| Rps19-ps7 | 3.25 | 0.09951103 |
| Rab20 | 3.23504274 | 0.00069057 |
| Gm10425 | 3.23076923 | 0.01770256 |
| Lrrc63 | 3.22222222 | 0.02653633 |
| Fpr1 | 3.2 | 0.22538845 |
| Ednrb | 3.17721519 | 0.00884924 |
| Gadd45a | 3.08494784 | 6.0359E-05 |
| Fas | 3.07647059 | 0.00274732 |
| Gm17024 | 3.07272727 | 0.00078964 |
| Ppp1r15a | 3.04568528 | 0.00172539 |
| Birc3 | 3.03432902 | 2.4407E-05 |
| Exoc3l4 | 3.03030303 | 0.01410884 |
| Gm6377 | 3.02147971 | 0.00022102 |
| Cdc42ep4 | 3.01512374 | 0.00019238 |
| Cited2 | 3.00597692 | 4.9235E-05 |
| Ltb4r1 | 3 | 0.17552337 |
| RP23-138K22.4 | 3 | 0.43450362 |
| Gm12571 | 3 | 0.27943964 |
| Gm16364 | 3 | 0.07121018 |
| Efna2 | 2.98039216 | 0.00022661 |
| Gm20100 | 2.94736842 | 0.07733809 |
| Nupr1 | 2.91256831 | 0.00114638 |
| Tnfrsf8 | 2.89473684 | 0.02422859 |
| Tnip1 | 2.88300443 | 0.00031775 |
| Gm42725 | 2.875 | 0.02200287 |
| Hspa1a | 2.84302326 | 0.00031905 |
| Spint2 | 2.83333333 | 0.04798336 |
| 5930403N24Rik | 2.83333333 | 0.02160799 |
| H2-M2 | 2.82857143 | 0.00892431 |
| E230016M11Rik | 2.8245614 | 0.00030599 |
| Cebpb | 2.8217233 | 3.1039E-05 |
| F3 | 2.8 | 0.09444633 |
| Dusp5 | 2.78380144 | 0.00043473 |
| Gm8818 | 2.77777778 | 0.16327899 |
| Trank1 | 2.77777778 | 0.28947747 |
| Gadd45b | 2.77391604 | 2.9349E-05 |
| Nfkbid | 2.75873434 | 0.00016957 |
| Gm43445 | 2.75 | 0.00101666 |
| Gm26522 | 2.75 | 0.16478902 |
| Gm14306 | 2.75 | 0.21197086 |
| Gm19026 | 2.75 | 0.04692107 |
| Stx11 | 2.73496659 | 0.00035875 |
| Gm16161 | 2.72972973 | 0.0152982 |
| Marcksl1 | 2.71348016 | 0.0001741 |
| Map3k8 | 2.68316832 | 0.00045594 |
| Sema3f | 2.66666667 | 0.48825088 |
| Oaf | 2.65207877 | 0.00034921 |
| Zfp36 | 2.63369668 | 0.00064642 |
| Saa3 | 2.61971831 | 0.03264828 |
| Il4i1 | 2.61904762 | 0.00766307 |
| Gm20481 | 2.61111111 | 0.049692 |
| Bcar1 | 2.61 | 0.00047629 |
| Fcrlb | 2.6 | 0.02893715 |
| Tnni3k | 2.6 | 0.48810281 |
| Csrnp1 | 2.59895151 | 0.00018352 |
| Rnf225 | 2.58333333 | 0.0902321 |
| Hcar2 | 2.58333333 | 0.00043409 |
| Gata2 | 2.58024691 | 0.00147508 |
| Rasd2 | 2.57142857 | 0.15697509 |
| Gm10284 | 2.57142857 | 0.14481833 |
| Rps12-ps26 | 2.57142857 | 0.34552387 |
| Gm23969 | 2.55555556 | 0.11611652 |
| Ccl22 | 2.54166667 | 0.05123084 |
| Gm6142 | 2.53846154 | 0.1200789 |
| Gm11707 | 2.53846154 | 0.01301195 |
| Ifi205 | 2.53149606 | 0.0049374 |
| Ccpg1os | 2.52631579 | 0.01584371 |
| Bcl2a1d | 2.52592593 | 0.00409134 |
| H2-Q5 | 2.51694915 | 0.01592197 |
| Rhbdl1 | 2.5 | 0.07172333 |
| Rps8-ps4 | 2.5 | 0.35738691 |
| RP24-233B16.1 | 2.5 | 0.03609766 |
| Sowahc | 2.48574238 | 2.9166E-05 |
| Spata13 | 2.48124718 | 6.8918E-05 |
| Rnf19b | 2.48120301 | 1.6585E-05 |
| Errfi1 | 2.4713217 | 6.9578E-05 |
| Kazald1 | 2.46666667 | 0.06856359 |
| Cd40 | 2.45994065 | 0.00184113 |
| Sema4b | 2.44642857 | 0.00089584 |
| Tnfrsf9 | 2.4375 | 0.08293904 |
| D930030I03Rik | 2.4375 | 0.08293904 |
| Rab11fip1 | 2.41729011 | 2.8023E-05 |
| Ahr | 2.40963855 | 0.01626944 |
| Ptges | 2.40686275 | 0.00063447 |
| Egfem1 | 2.4 | 0.06574285 |
| Sfmbt2 | 2.38461538 | 0.12423598 |
| Plpp3 | 2.38461538 | 0.01024523 |
| Immp2l | 2.38095238 | 0.25410364 |
| Lrrc32 | 2.36363636 | 0.07172333 |
| Gm37716 | 2.33333333 | 0.15830242 |
| Gm20655 | 2.33333333 | 0.011355 |
| Mmp25 | 2.31578947 | 0.00971665 |
| Src | 2.30769231 | 0.00920589 |
| Gm17083 | 2.3 | 0.12157921 |
| Gm14233 | 2.29032258 | 0.03737084 |
| Cx3cl1 | 2.28834356 | 0.00054486 |
| Gm44270 | 2.28571429 | 0.31680156 |
| Gm44662 | 2.28571429 | 0.36671548 |
| Gm28592 | 2.28227571 | 0.00204598 |
| Six1 | 2.27272727 | 0.00535882 |
| Slfn2 | 2.25812285 | 0.00016202 |
| Htra1 | 2.25 | 0.05197969 |
| Gm10059 | 2.25 | 0.06676654 |
| Relb | 2.24977659 | 0.0009136 |
| Trim13 | 2.24409449 | 0.00025794 |
| 4933416M07Rik | 2.23225806 | 0.00198944 |
| 1110002J07Rik | 2.23019802 | 0.00287867 |
| Gm43305 | 2.22692308 | 0.00046123 |
| Snora62 | 2.22222222 | 0.23178577 |
| Clec2d | 2.22024867 | 0.00108866 |
| RP23-392I13.15 | 2.21875 | 0.00047858 |
| Sdc4 | 2.21760714 | 4.0113E-05 |
| F10 | 2.2173913 | 0.11611652 |
| Gm6473 | 2.21052632 | 0.2239482 |
| Il10ra | 2.20771001 | 4.4251E-06 |
| Gm37592 | 2.20689655 | 0.22972795 |
| Timp1 | 2.2 | 0.05248879 |
| Gm13657 | 2.2 | 0.20218564 |
| Tnfrsf1b | 2.19544294 | 6.1746E-05 |
| Cfap73 | 2.1875 | 0.25322682 |
| 4930440I19Rik | 2.1875 | 0.07331606 |
| Gm20186 | 2.18644068 | 0.01216101 |
| 1700086L19Rik | 2.18181818 | 0.06004813 |
| Gm8515 | 2.18181818 | 0.18743651 |
| Gm16106 | 2.18181818 | 0.04064209 |
| Phlda1 | 2.15194805 | 0.0004897 |
| RP24-167A12.4 | 2.15 | 0.02843926 |
| 2500002B13Rik | 2.14814815 | 0.00011344 |
| Mllt6 | 2.14589199 | 0.00024504 |
| Herpud1 | 2.13899254 | 0.00092441 |
| Fam83c | 2.13793103 | 0.00672707 |
| Rasgrp2 | 2.125 | 0.05333826 |
| Mxd1 | 2.12096774 | 0.00125679 |
| Fxyd3 | 2.11764706 | 0.03112563 |
| Gm14154 | 2.11111111 | 0.29801481 |
| Gm26576 | 2.11111111 | 0.05015089 |
| Gm9845 | 2.10526316 | 0.31894957 |
| Il17c | 2.10526316 | 0.11219398 |
| Rpl7-ps7 | 2.1 | 0.03654829 |
| 3110043O21Rik | 2.09894951 | 0.00020981 |
| Gfi1b | 2.09090909 | 0.00105756 |
| Icam5 | 2.09090909 | 0.11242104 |
| Bcat1 | 2.09090909 | 0.18407403 |
| Gm43912 | 2.08888889 | 0.06139562 |
| Phldb1 | 2.08752556 | 0.0011835 |
| Gm43963 | 2.08518519 | 0.00083946 |
| Lamc2 | 2.08510638 | 0.05392681 |
| Rtn2 | 2.08333333 | 0.12672652 |
| Acpp | 2.08333333 | 0.04541277 |
| Gm13807 | 2.07692308 | 0.40443286 |
| Orai2 | 2.06832716 | 0.00030691 |
| Ehd1 | 2.05753177 | 0.00016504 |
| Spaca6 | 2.04861111 | 0.00571936 |
| Ccdc92 | 2.04761905 | 0.04798336 |
| Gm8325 | 2.04651163 | 0.50257252 |
| Gm21887 | 2.04560261 | 0.35098461 |
| Zswim4 | 2.0391523 | 0.00010405 |
| Rnd3 | 2.03060109 | 0.00017189 |
| Pdgfb | 2.01748204 | 0.00017516 |
| Gm28913 | 2.00680272 | 0.0036922 |
| RP23-371B13.3 | 2 | 0.46970612 |
| Bspry | 2 | 0.23810831 |
| Gm29257 | 2 | 0.33006565 |
| Rhobtb3 | 2 | 0.21181611 |
| Gm5456 | 2 | 0.16710332 |
| Fdx1l | 2 | 0.13041164 |
| Gm12216 | 2 | 0.18716367 |
| Olfr1444 | 2 | 0.00653338 |
| Gm43011 | 2 | 0.09406601 |
| B430010I23Rik | 2 | 0.0005985 |
| Gstt1 | 2 | 0.13063511 |
| Slc6a4 | 2 | 0.36708617 |
| Hbegf | 2 | 0.21620474 |

**Supplemental material 3b: Table of downregulated RNAs in macrophages by skeletal muscle EVs**

| Gene | Fold change | P-value |
| --- | --- | --- |
| Mip | 0.05263158 | 0.03347174 |
| Gm4849 | 0.09090909 | 0.04336034 |
| Gm15950 | 0.09090909 | 0.04742066 |
| 6030400A10Rik | 0.13461538 | 0.04765593 |
| Dlx2 | 0.14285714 | 0.00359311 |
| Zfp791 | 0.1637931 | 0.00038082 |
| Hes1 | 0.16666667 | 0.00749043 |
| Gm37669 | 0.19047619 | 0.03250537 |
| Gm22358 | 0.19191919 | 0.00812086 |
| Ier5l | 0.22710623 | 2.7643E-05 |
| Cbx8 | 0.2437276 | 0.0010499 |
| Gm10384 | 0.25 | 0.01764102 |
| Heg1 | 0.25 | 0.02131164 |
| Gm15489 | 0.25 | 0.04231566 |
| Hist1h2bj | 0.25 | 0.04994809 |
| Ano7 | 0.25714286 | 0.00119288 |
| Slc16a5 | 0.26666667 | 0.00197091 |
| Gm13071 | 0.26666667 | 0.04798336 |
| Dusp6 | 0.26963461 | 0.00013577 |
| H3f3aos | 0.27586207 | 0.00318481 |
| 4732463B04Rik | 0.27586207 | 0.02380611 |
| Cpsf4l | 0.27586207 | 0.02380611 |
| Thbd | 0.28444084 | 4.0761E-05 |
| Jph3 | 0.28571429 | 0.04742066 |
| Chrna2 | 0.29411765 | 0.03267792 |
| Insl3 | 0.3 | 0.02166358 |
| RP23-423B21.6 | 0.30188679 | 0.00820571 |
| Adrb1 | 0.30434783 | 0.00064544 |
| Gm11527 | 0.30434783 | 0.02321516 |
| Lpar6 | 0.31103388 | 3.0785E-05 |
| Nap1l5 | 0.32608696 | 0.03786056 |
| 1700020D05Rik | 0.34285714 | 0.01941607 |
| Gm10478 | 0.34615385 | 0.00920589 |
| Rgs2 | 0.34893758 | 2.0992E-05 |
| Id1 | 0.35604686 | 0.00021713 |
| Gm20658 | 0.36206897 | 0.01321922 |
| BC043934 | 0.36486486 | 0.0093084 |
| BC085271 | 0.36585366 | 0.00517963 |
| Zfp273 | 0.36619718 | 0.01611684 |
| 1700012D14Rik | 0.37254902 | 0.00537044 |
| Gm37447 | 0.375 | 0.00749043 |
| Gm807 | 0.375 | 0.03759011 |
| Gm26735 | 0.38166311 | 0.00326454 |
| Letm2 | 0.38461538 | 0.02321516 |
| Gm14288 | 0.3852459 | 0.02952816 |
| A130014A01Rik | 0.38888889 | 0.00493352 |
| Dnm1 | 0.39130435 | 0.04881155 |
| Rps26-ps1 | 0.39179954 | 0.00695623 |
| Frat1 | 0.39215686 | 0.02074071 |
| Gm12258 | 0.39269406 | 2.5889E-05 |
| Zfp239 | 0.39393939 | 0.00164129 |
| AV356131 | 0.39393939 | 0.03410942 |
| 2010015M23Rik | 0.39534884 | 0.02126075 |
| Fosb | 0.3976915 | 0.00616517 |
| 3300005D01Rik | 0.4 | 0.0050268 |
| Col7a1 | 0.4 | 0.04231566 |
| Rhov | 0.40066593 | 0.00150817 |
| Osgin1 | 0.40214067 | 6.2554E-05 |
| A430110L20Rik | 0.40816327 | 0.00685502 |
| Rab3a | 0.40948276 | 0.00215963 |
| Gm44974 | 0.41025641 | 0.01218966 |
| Prx | 0.41304348 | 0.01788717 |
| Cd46 | 0.41489362 | 0.00322756 |
| Arc | 0.41772152 | 0.02962249 |
| Arrdc3 | 0.41924227 | 0.00028456 |
| Mafb | 0.42008768 | 2.2358E-05 |
| Gm29666 | 0.42222222 | 0.022646 |
| Gm30238 | 0.42241379 | 0.04044258 |
| Sox4 | 0.42475387 | 0.00061325 |
| Hhex | 0.42729767 | 0.00019999 |
| Gm10509 | 0.42857143 | 0.02306381 |
| Olfr99 | 0.4375 | 0.02422859 |
| Inpp5j | 0.44444444 | 0.00516687 |
| Gpr146 | 0.44570502 | 3.5911E-05 |
| A630035G10Rik | 0.44827586 | 0.01783265 |
| C630043F03Rik | 0.44927536 | 0.00199149 |
| A930024E05Rik | 0.4527027 | 0.01128629 |
| Gm38289 | 0.45454545 | 0.00048413 |
| Hmgb1-ps8 | 0.45490196 | 0.03550459 |
| 9930012K11Rik | 0.4556962 | 0.00215313 |
| Zfp61 | 0.46190476 | 0.00640019 |
| Aqp11 | 0.46341463 | 0.0177043 |
| 3222401L13Rik | 0.47058824 | 0.01407448 |
| Frat2 | 0.47222222 | 0.02594656 |
| Slc16a4 | 0.47297297 | 0.0261225 |
| Zfp128 | 0.47337278 | 0.01772088 |
| Fam78a | 0.48433243 | 0.00074109 |
| Slc6a19 | 0.48484848 | 0.01421686 |
| Gm28626 | 0.49206349 | 0.02172018 |
| Gm15774 | 0.49206349 | 0.0405774 |
| Gm13179 | 0.49264706 | 0.02523651 |
| 6330409D20Rik | 0.49275362 | 0.00831755 |
| Gm45110 | 0.5 | 0.02720977 |
| Gm44698 | 0.5 | 0.03389835 |
| Mfsd4b3 | 0.5 | 0.0367662 |
