# Supplementary material for: Skeletal myotube-derived extracellular vesicles enhance itaconate production and attenuate inflammatory responses of macrophages
Source: Front Immunol. 2023 Mar 2;14:1099799. doi: 10.3389/fimmu.2023.1099799 (PMC10018131; doi:10.3389/fimmu.2023.1099799)
Supplement: Supplementary file 1 [file DataSheet_1.zip › Supplemental material 4.DOCX]

**Supplemental material 4: List of miRNAs detected in skeletal myotube-derived extracellular vesicles**

| miRNAs |
| --- |
| miR-206-3p |
| miR-378a-3p |
| miR-30d-5p |
| miR-21a-5p |
| miR-99a-5p |
| miR-128-3p |
| miR-148a-3p |
| miR-140-3p |
| miR-532-5p |
| miR-30a-5p |
| miR-128-3p |
| miR-1a-3p |
| miR-143-3p |
| miR-27a-3p |
| let-7i-5p |
| let-7f-5p |
| let-7c-5p |
| let-7c-5p |
| let-7f-5p |
| miR-22-3p |
| miR-221-3p |
| miR-27b-3p |
| miR-182-5p |
| let-7b-5p |
| miR-320-3p |
| miR-501-3p |
| miR-25-3p |
| miR-99b-5p |
| miR-29a-3p |
| let-7g-5p |
| miR-222-3p |
| miR-191-5p |
| miR-151-3p |
| miR-183-5p |
| miR-26a-5p |
| miR-26a-5p |
| miR-149-5p |
| miR-152-3p |
| miR-100-5p |
| let-7a-5p |
| let-7a-5p |
| miR-192-5p |
| miR-23a-3p |
| let-7d-3p |
| miR-322-3p |
| miR-378c |
| miR-674-3p |
| miR-22-5p |
| miR-139-5p |
| miR-423-3p |
| miR-1839-5p |
| miR-615-3p |
| miR-99b-3p |
| miR-1198-5p |
| let-7d-5p |
| miR-30c-5p |
| miR-30c-5p |
| miR-199a-3p |
| miR-199a-3p |
| miR-199b-3p |
| miR-214-3p |
| miR-378b |
| miR-30a-3p |
| miR-503-3p |
| miR-185-5p |
| miR-196a-5p |
| miR-125a-5p |
| miR-30e-5p |
| miR-196a-5p |
| miR-298-5p |
| miR-328-3p |
| miR-181d-5p |
| miR-224-5p |
| miR-1981-5p |
| miR-362-3p |
| miR-199a-5p |
| miR-199a-5p |
| let-7e-5p |
| miR-744-5p |
| miR-1964-3p |
| miR-133a-3p |
| miR-133a-3p |
| miR-423-5p |
| miR-92a-3p |
| miR-484 |
| miR-28a-3p |
| miR-361-3p |
| miR-184-3p |
| miR-181a-5p |
| miR-181a-5p |
| miR-499-5p |
| miR-125b-5p |
| miR-125b-5p |
| miR-351-5p |
| miR-127-3p |
| miR-181b-5p |
| miR-125b-1-3p |
| miR-181b-5p |
| miR-26b-5p |
| miR-145a-3p |
| miR-872-5p |
| miR-451a |
| miR-345-3p |
| miR-125a-3p |
| miR-1843a-5p |
| miR-218-5p |
| miR-7a-5p |
| miR-218-5p |
| miR-101a-3p |
| miR-7a-5p |
| miR-34b-3p |
| miR-133b-3p |
| miR-10a-5p |
| miR-148b-3p |
| miR-381-3p |
| miR-1843b-5p |
| miR-378d |
| miR-101b-3p |
| miR-23b-3p |
| miR-574-3p |
| miR-34c-3p |
| let-7a-1-3p |
| let-7c-2-3p |
| miR-10b-5p |
| miR-103-3p |
| miR-98-5p |
| miR-103-3p |
| miR-30b-5p |
| miR-146b-5p |
| miR-186-5p |
| miR-34c-5p |
| miR-434-5p |
| miR-31-5p |
| miR-132-3p |
| miR-542-3p |
| miR-93-5p |
| miR-1968-5p |
| miR-214-5p |
| miR-140-5p |
| miR-664-5p |
| miR-125b-2-3p |
| miR-425-5p |
| miR-331-5p |
| miR-455-5p |
| miR-133a-5p |
| miR-20a-5p |
| miR-330-3p |
| miR-3057-5p |
| miR-145a-5p |
| miR-503-5p |
| miR-671-3p |
| miR-196b-5p |
| miR-133a-5p |
| miR-708-3p |
| miR-342-3p |
| miR-106b-3p |
| miR-351-3p |
| miR-146a-5p |
| miR-365-2-5p |
| miR-339-3p |
| miR-130a-3p |
| miR-200b-3p |
| miR-335-5p |
| miR-152-5p |
| miR-296-3p |
| let-7f-1-3p |
| miR-210-3p |
| miR-500-3p |
| miR-27a-5p |
| miR-92a-3p |
| miR-199b-5p |
| miR-6240 |
| miR-598-3p |
| miR-379-5p |
| miR-5099 |
| miR-3068-3p |
| miR-16-5p |
| miR-16-5p |
| miR-194-5p |
| miR-17-5p |
| miR-194-5p |
| miR-362-5p |
| miR-155-5p |
| miR-132-5p |
| miR-370-3p |
| miR-330-5p |
| miR-421-3p |
| miR-361-5p |
| miR-452-5p |
| miR-7666-3p |
| miR-5121 |
| miR-148b-5p |
| miR-200c-3p |
| miR-340-5p |
| miR-200a-3p |
| miR-188-5p |
| miR-96-5p |
| miR-6944-3p |
| miR-5114 |
| miR-760-3p |
| miR-1983 |
| miR-339-5p |
| miR-541-5p |
| let-7b-3p |
| miR-700-3p |
| miR-344d-3p |
| miR-344d-3p |
| miR-532-3p |
| miR-344d-3p |
| miR-450b-3p |
| miR-1943-5p |
| miR-6540-5p |
| miR-411-5p |
| miR-335-3p |
| miR-483-5p |
| miR-106b-5p |
| miR-107-3p |
| miR-378a-5p |
| miR-27b-5p |
| miR-1298-5p |
| miR-129-5p |
| miR-3535 |
| miR-3102-5p.2-5p |
| miR-193a-5p |
| miR-1945 |
| miR-7680-5p |
| miR-1839-3p |
| miR-300-3p |
| miR-7680-3p |
| miR-202-5p |
| miR-652-3p |
| miR-434-3p |
| miR-212-5p |
| miR-191-3p |
| miR-877-5p |
| miR-28a-5p |
| miR-3095-3p |
| miR-425-3p |
| let-7e-3p |
| miR-1843b-3p |
| miR-204-5p |
| miR-34a-5p |
| miR-350-5p |
| miR-143-5p |
| miR-465c-5p |
| miR-465c-5p |
| miR-215-5p |
| miR-98-3p |
| miR-409-3p |
| miR-7068-3p |
| miR-674-5p |
| miR-881-3p |
| miR-7689-3p |
| miR-3081-3p |
| miR-6988-3p |
| miR-128-2-5p |
| miR-30c-2-3p |
| miR-669c-5p |
| miR-29b-3p |
| miR-29b-3p |
| miR-878-5p |
| miR-6516-5p |
| miR-450a-5p |
| miR-450a-5p |
| miR-34b-5p |
| miR-148a-5p |
| miR-664-3p |
| miR-470-5p |
| miR-582-3p |
| miR-130b-5p |
| miR-3099-3p |
| miR-341-3p |
| miR-187-3p |
| miR-23b-5p |
| miR-3474 |
| miR-222-5p |
| miR-375-3p |
| miR-6911-3p |
| miR-195a-3p |
| miR-127-5p |
| miR-6929-3p |
| miR-142a-5p |
| miR-671-5p |
| miR-221-5p |
| let-7c-1-3p |
| miR-677-5p |
| miR-1291 |
| miR-872-3p |
| miR-29c-5p |
| miR-1249-3p |
| miR-1306-3p |
| miR-6979-3p |
| miR-323-3p |
| miR-200a-5p |
| miR-1199-5p |
| miR-6952-3p |
| miR-223-3p |
| miR-6896-5p |
| miR-675-3p |
| miR-382-3p |
| miR-6948-3p |
| miR-495-3p |
| miR-92b-3p |
| miR-543-3p |
| miR-29a-5p |
| miR-205-5p |
| miR-331-3p |
| miR-1231-5p |
| miR-6997-5p |
| miR-15b-3p |
| let-7f-2-3p |
| miR-181a-2-3p |
| miR-31-3p |
| miR-1941-3p |
| miR-151-5p |
| miR-139-3p |
| miR-429-3p |
| miR-7b-5p |
| miR-30b-3p |
| miR-6966-3p |
| miR-16-1-3p |
| miR-181c-5p |
| miR-877-3p |
| miR-181c-3p |
| miR-329-3p |
| miR-32-5p |
| miR-410-3p |
| miR-19a-3p |
| miR-8103 |
| miR-185-3p |
| miR-190b-5p |
| miR-322-5p |
| miR-704 |
| miR-6928-3p |
| miR-6956-3p |
| miR-208b-3p |
| miR-1981-3p |
| miR-1191b-5p |
| miR-350-3p |
| miR-363-3p |
| miR-5129-3p |
| miR-383-5p |
| miR-193b-3p |
| miR-195a-5p |
| miR-329-5p |
| miR-1960 |
| miR-1843a-3p |
| miR-3068-5p |
| miR-134-5p |
| miR-547-3p |
| miR-3102-3p |
| miR-1947-5p |
| miR-6900-3p |
| miR-433-3p |
| miR-21a-3p |
| miR-409-5p |
| miR-369-3p |
| miR-182-3p |
| miR-431-3p |
| miR-344-3p |
| miR-1941-5p |
| miR-187-5p |
| miR-3097-3p |
| miR-6958-3p |
| miR-196a-2-3p |
| miR-501-5p |
| miR-29c-3p |
| miR-344-3p |
| miR-1934-5p |
| miR-8094 |
| miR-666-5p |
| miR-3064-5p |
| miR-494-3p |
| miR-10a-3p |
| miR-382-5p |
| miR-744-3p |
| miR-181a-1-3p |
| miR-19b-3p |
| miR-19b-3p |
| miR-328-5p |
| miR-488-3p |
| miR-133b-5p |
| miR-431-5p |
| miR-7a-1-3p |
| miR-365-3p |
| miR-365-3p |
| miR-12192-5p |
| miR-30d-3p |
| miR-3058-3p |
| miR-6948-5p |
| miR-5113 |
| miR-615-5p |
| miR-7648-3p |
| miR-342-5p |
| miR-8120 |
| miR-223-5p |
| miR-212-3p |
| miR-345-5p |
| miR-344b-3p |
| miR-153-3p |
| miR-7021-5p |
| miR-741-3p |
| miR-7075-3p |
| let-7i-3p |
| miR-138-2-3p |
| miR-8114 |
| miR-3061-3p |
| miR-8105 |
| miR-376b-5p |
| miR-12194-3p |
| miR-135b-5p |
| miR-871-3p |
| miR-324-5p |
| miR-12181-3p |
| miR-147-3p |
| miR-380-3p |
| miR-124-3p |
| miR-124-3p |
| miR-124-3p |
| miR-210-5p |
| miR-23a-5p |
| miR-1949 |
| miR-504-5p |
| miR-7652-3p |
| miR-708-5p |
| miR-6903-3p |
| miR-540-3p |
| miR-1934-3p |
| miR-5132-5p |
| miR-465a-5p |
| miR-153-5p |
| miR-6916-5p |
| miR-21b |
| miR-542-5p |
| miR-675-5p |
| miR-3473g |
| miR-7008-3p |
| miR-301a-3p |
| miR-7667-3p |
| miR-6516-3p |
| miR-3094-3p |
| miR-135a-2-3p |
