# Supplementary material for: Skeletal myotube-derived extracellular vesicles enhance itaconate production and attenuate inflammatory responses of macrophages
Source: Front Immunol. 2023 Mar 2;14:1099799. doi: 10.3389/fimmu.2023.1099799 (PMC10018131; doi:10.3389/fimmu.2023.1099799)
Supplement: Supplementary file 1 [file DataSheet_1.zip › Supplemental material 5.PPTX]

## Slide 1
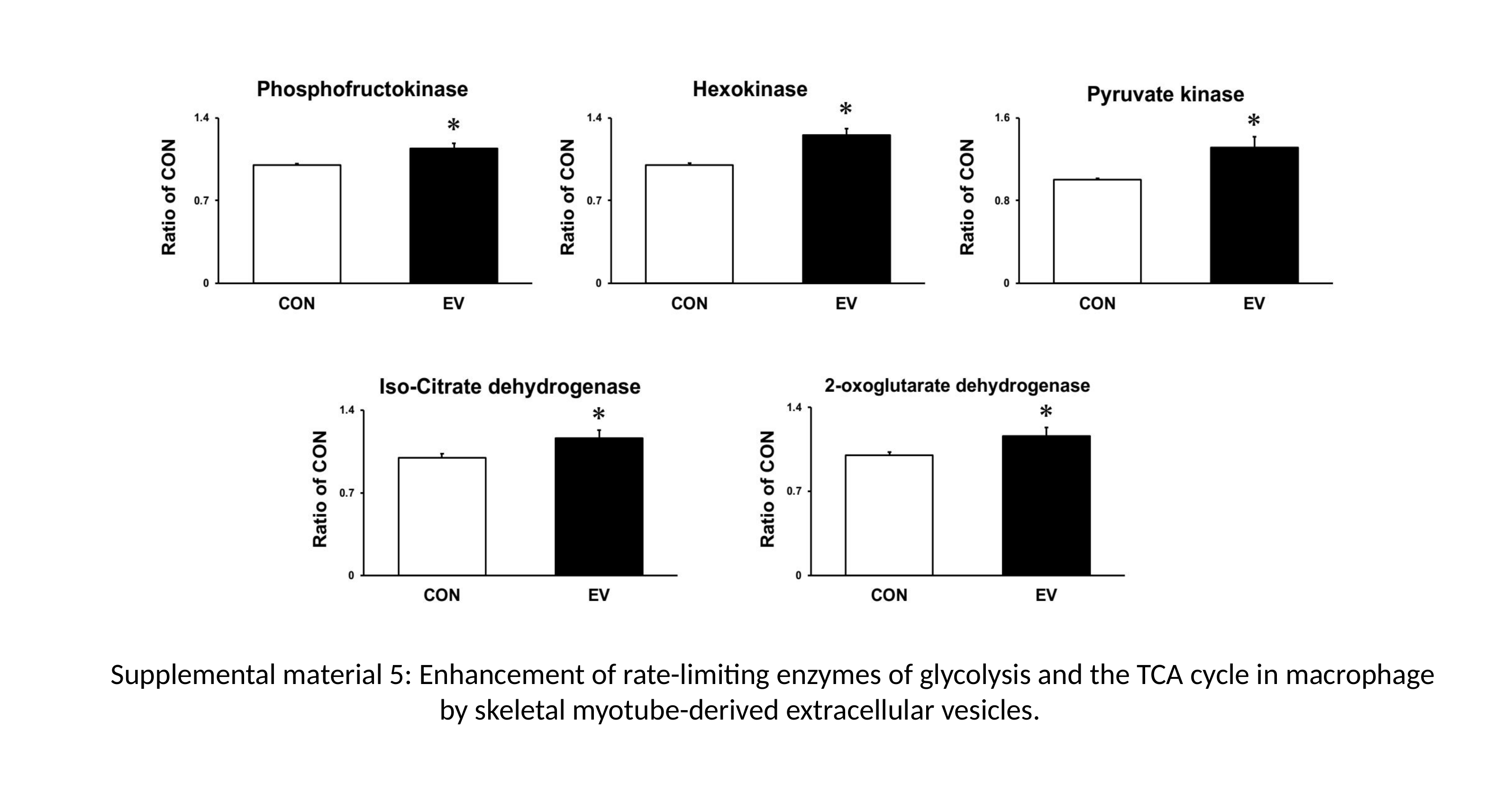

Supplemental material 5: Enhancement of rate-limiting enzymes of glycolysis and the TCA cycle in macrophage
　　　　　　　　 by skeletal myotube-derived extracellular vesicles.
